# Localization and Functional Characterization of a Novel Adipokinetic Hormone in the Mollusk, *Aplysia californica*

**DOI:** 10.1371/journal.pone.0106014

**Published:** 2014-08-27

**Authors:** Joshua I. Johnson, Scott I. Kavanaugh, Cindy Nguyen, Pei-San Tsai

**Affiliations:** Department of Integrative Physiology and Center for Neuroscience, University of Colorado, Boulder, Colorado, United States of America; John Hopkins University School of Medicine, United States of America

## Abstract

Increasing evidence suggests that gonadotropin-releasing hormone (GnRH), corazonin, adipokinetic hormone (AKH), and red pigment-concentrating hormone all share common ancestry to form a GnRH superfamily. Despite the wide presence of these peptides in protostomes, their biological effects remain poorly characterized in many taxa. This study had three goals. First, we cloned the full-length sequence of a novel AKH, termed *Aplysia*-AKH, and examined its distribution in an opisthobranch mollusk, *Aplysia californica*. Second, we investigated *in vivo* biological effects of *Aplysia*-AKH. Lastly, we compared the effects of *Aplysia*-AKH to a related *A. californica* peptide, *Aplysia*-GnRH. Results suggest that *Aplysia*-AKH mRNA and peptide are localized exclusively in central tissues, with abdominal, cerebral, and pleural ganglia being the primary sites of *Aplysia*-AKH production. However, *Aplysia*-AKH-positive fibers were found in all central ganglia, suggesting diverse neuromodulatory roles. Injections of *A. californica* with *Aplysia*-AKH significantly inhibited feeding, reduced body mass, increased excretion of feces, and reduced gonadal mass and oocyte diameter. The *in vivo* effects of *Aplysia*-AKH differed substantially from *Aplysia*-GnRH. Overall, the distribution and biological effects of *Aplysia*-AKH suggest it has diverged functionally from *Aplysia*-GnRH over the course of evolution. Further, that both *Aplysia*-AKH and *Aplysia*-GnRH failed to activate reproduction suggest the critical role of GnRH as a reproductive activator may be a phenomenon unique to vertebrates.

## Introduction

Adipokinetic hormone and red pigment-concentrating hormones (AKH/RPCHs) are structurally related peptides with well-characterized functions in arthropods [1]. Recently it has been shown that corazonin (CRZ) and gonadotropin-releasing hormone (GnRH) likely share common ancestry with AKH/RPHC peptides [2], thus forming a GnRH superfamily. In vertebrates, GnRH activates the hypothalamo-pituitary-gonadal (HPG) axis to initiate reproduction. AKH and CRZ are considered as stress hormones [3], [4] and are released from the corpora cardiaca (CC) in insects, which is an organ innervated by neurosecretory cells in the protocerebrum [5]–[7]. Similarly, RPCHs, a group of multifunctional crustacean neuropeptides, are synthesized in neurons of the X-organ (XO) and then transported to the sinus gland (SG) to be stored and released [7]. Both the protocerebrum/CC and XO-SG are neuroendocrine complexes considered to be analogous to the vertebrate hypothalamo-hypophysial system [8]–[10]. Although these peptides are structurally and evolutionarily related [2], their functions vary somewhat across and within phyla.

Metabolic involvement in some capacity is a common functional theme for many members of the GnRH superfamily. The most well-described function of AKH is the mobilization of energy stores, including carbohydrates, lipids, and proteins, during energy-intense activities in insects [4], [11]. RPCH, although not known for its metabolic involvement, has been shown to modulate the stomatogastric nervous system in crustaceans [12]–[14], which is involved in the control of gut motility [15]. CRZ was first discovered as a cardioacceleratory peptide [16] but is now hypothesized to covey nutritional stress signals from a poor diet [3]. Finally, GnRH-II, a form of GnRH found in the midbrain of many vertebrates [17], is involved in the regulation of feeding [18]–[20].

Ties to reproductive physiology are also found within the GnRH superfamily. In addition to the well-established role of vertebrate GnRH in HPG axis activation, other members of the superfamily also exhibit reproductive effects. For example, CRZ inhibits the androgenic gland, testicular development, and spermatogenesis in the prawn, *Macrobrachium rosenbergii* [21], [22]. Evidence from the cricket [23] and the locust [24] suggests that AKH may inhibit female insect reproduction. Conversely, RPCH and GnRH have been shown to stimulate ovarian maturation in the crayfish, *Procambarus clarkii* [25], [26].

Until recently, little was known about the function of AKH/RPCH in non-arthropod protostomes. In 2011, the first sequences encoding molluscan AKH, one in the sea hare (*Aplysia californica*) and another in the owl limpet (*Lottia gigantea*), were identified by data mining [2]. However, the molecular and functional nature of these two molluscan AKH had not been explored further. The primary goals of the present study were to identify the site of AKH synthesis and examine its biological activities in *A. californica*. To accomplish these goals, we first authenticated *Aplysia* AKH (ap-AKH) by molecular cloning and localized mature peptide and transcript of ap-AKH in *A. californica* central ganglia using immunocytochemistry (ICC) and *in situ* hybridization (ISH), respectively. Next, we functionally characterized ap-AKH *in vivo* by examining its effects on several physiological parameters including metabolism and reproduction. Lastly, we compared the biological activities of ap-AKH to a related peptide named *Aplysia* GnRH (ap-GnRH). As a protostome known to simultaneously express an AKH and a GnRH homolog, *A. californica* represents an excellent model for understanding the functional divergence of two evolutionarily related peptides. Our results show that ap-AKH induces changes in diverse physiological parameters such as feeding, volume regulation, hemolymph glucose, and gut activity, but inhibited several gonadal parameters. Importantly, the biological effects of ap-AKH have diverged substantially from those of ap-GnRH [27]. These data demonstrate that protostomian members of the GnRH superfamily are surprisingly versatile in their biological activities and suggest the trajectory of functional evolution for these peptides may be extremely complex.

## Materials and Methods

### 1. Animals

Wild-caught *A. californica* (100–300 g) were purchased from Alacrity Marine Biological Services (Redondo Beach, CA). Animals were housed individually in perforated floating cages in 200 gallons of artificial seawater (Instant Ocean, Cincinnati, OH). The salinity was monitored daily and maintained between 1020 and 1023 kg/m^3^. Water was maintained at 18–20°C, aerated, and re-circulated through biological and chemical filters. *A. californica* processed for ICC/ISH were fed a diet of Kale daily, whereas those used for injection studies were fed a diet of Romaine lettuce. No difference in the health status was observed between animals fed exclusively on either diet. No institutional approval was required for the use of invertebrates such as *A. californica*, but the authors adhered to standards of humane treatment to minimize pain, stress, and suffering of experimental animals.

### 2. Cloning of ap-AKH

Guided by the ap-AKH peptide sequence described by Roch et al. [2], we performed tblastn search of *A. californica* expressed sequence tags. The query resulted in several sequences that were used to design a forward and a reverse gene-specific primer (GSP) for the 5′ and 3′ rapid amplification of cDNA ends (RACE) of *prepro ap-AKH*. RACE was performed using the GeneRacer kit (Invitrogen, Grand Island, NY) according to manufacturer’s instructions. Briefly, cDNA synthesized from the central nervous system (CNS) total RNA was amplified using *ap-AKH* GSPs (forward primer, 5′-ATGGAATCTTCTAGCATACTTTTG-3′; reverse primer, 5′-CAGTTCTGGGCAGGTATTCAGG-3′) in conjunction with 5′ and 3′ RACE adapter primers from the GeneRacer kit to obtain overlapping 5′ and 3′ sequences of *prepro ap-AKH*. Amplicons were subcloned into pGEM T-Easy (Promega, Madison, WI) and sequenced to deduce the full-length *prepro ap-AKH* cDNA sequence, including the 5′ and 3′ untranslated regions (UTR).

### 3. Reverse-transcription polymerase chain reaction (RT-PCR)

Total RNA was isolated from bag cell neurons (BCN; two discrete clusters of neuroendocrine neurons anterior to the abdominal ganglion), abdominal ganglion, cerebral ganglia, pedal/pleural ganglia, buccal ganglia, tail muscle, osphradium (a chemo- and osmosensory organ), small hermaphroditic duct (a gamete-transporting duct), heart, and ovotestis (OVT) using TRIzol Reagent (Invitrogen). RNA samples were pretreated with RQ1 DNase (Promega, Madison, WI) before RT reaction using the Superscript III first-strand cDNA synthesis kit (Invitrogen). *ap-AKH* was amplified using *ap-AKH* GSPs listed above, and *ap-Actin* amplified using specific actin primers (forward primer: 5′-GGTATTGTGTTGGACTCTGG-3′; reverse primer: 5′-TGATGGAGTTGAAGGTGGTC-3′). Parameters for PCR reactions were identical to those previously described [28].

### 4. Tissue harvest and preparation

*A. californica* were anesthetized with an injection of 1/3 body mass of ice-cold isotonic MgCl_2_. Central ganglia (buccal, cerebral, pedal, pleural, and abdominal) were harvested, individually pinned out on a Sylgard-lined dish, and fixed in 4% paraformaldehyde for 6 hours at 4°C. Tissues were then cryoprotected in 30% sucrose, embedded in OCT medium (Tissue-Tek, Torrance, CA), snap frozen on dry ice, and stored at −70°C until sectioning. Samples were sectioned at 12-µm thickness using a cryostat, and adjacent sections were thaw-mounted onto alternating slides to be processed for ICC and ISH separately. Because *A. californica* neurons were very large, ICC and ISH could be performed separately on adjacent sections containing the same neurons [29].

### 5. Immunocytochemistry

For ICC, a custom rabbit antiserum against ap-AKH was used (EZBiolab, Carmel, IN). The antiserum was generated against a synthetic ap-AKH (CIHFSPDWGT-amide) in which the N-terminal pyroglutamate was replaced with a cysteine residue for conjugation to keyhole limpet hemocyanin (KLH). The antiserum was preadsorbed overnight with 5 mg/ml KLH (Sigma-Aldrich, St. Louis, MO) to prevent crossreactivity with *A. californica* hemocyanin and used at 1∶1,000. ICC was carried out as previously described [29] and signals visualized with diaminobenzidine as the chromagen. After ICC, sections were counterstained with methyl green, dehydrated, and coverslipped. Specificity of ap-AKH staining was tested by preadsorbing the ap-AKH antiserum with 20 µg/ml synthetic ap-AKH peptide (pQIHFSPDWGT-amide; Genscript, Piscataway, NJ). ap-AKH-immunoreactive (ir) neurons were counted as previously described [29] to avoid counting the same neuron multiple times. ICC was performed on three separate *A. californica*, and each time yielded similar results.

### 6. *In situ* hybridization

A 243-nt antisense digoxigenin-labeled riboprobe was generated from the full-length cDNA of *ap-AKH* using the digoxigenin RNA labeling mix (Roche, Indianapolis, MN). The riboprobe was used at 200 ng/ml in a standard colorimetric ISH and visualized with nitro-blue-tetrazolium/5-bromo-4-chloro-3-indolyl-phosphate as previously described [29]. All sections were counterstained with methyl green, and neurons positive for *ap-AKH* were counted as described above for counting ap-AKH-ir neurons. ISH was performed on three separate *A. californica*, and each time yielded similar results.

### 7. ap-AKH injections

Upon arrival, all *A. californica* were acclimated to the laboratory environment and diet for at least 4 days. Synthetic ap-AKH peptide was prepared by diluting the concentrated peptide stock in sterile-filtered artificial seawater (ASW; 395 mM NaCl, 10 mM KCl, 10 mM CaCl_2_, 50 mM MgCl_2_, 28 mM Na_2_SO_4_, 30 mM HEPES) [30], [31] immediately prior to injections. All injections were 500 µl in volume and administered via 26-gauge needles through the posterior foot into the hemocoel. When food consumption was measured (Experiments 2 and 5), meal portions (∼7–13% body mass) and feeding durations (3 hours) were designed based on previously published studies [32]. The injection doses of ap-AKH were based on previously published ap-GnRH injection studies [27]. These doses were estimated to produce approximately 50 nM circulating peptide in the absence of protease degradation. The *in vitro* treatment of various forms of GnRH for the stimulation of gonadotropin secretion often fell within this range [33]–[35]. In our hands, injections rarely provoked inking, suggesting animals were not markedly stressed [36].

### 7.1. Experiment 1: acute effects of ap-AKH on hemolymph glucose levels

A well-known role of insect AKH is the elevation of circulating diacylglycerol or trehalose [7], [11]. The main circulating sugar in *A. californica* is glucose [32], [37], [38], thus ap-AKH was tested for acute hyperglycemic activity. All animals were food-deprived for 24 hours before the study. Baseline hemolymph samples were taken immediately prior to injection with either 30 µg ap-AKH or ASW vehicle (n = 5/group). Additional hemolymph samples were then taken at 10, 30, 60, and 120 minutes after injection. Approximately 500 µl hemolymph per animal was taken at each time point through the posterior foot using 22-gauge needles. According to our calculation, the hemolymph drawn within this 2-hour experimental period never exceeded 1.6% of the total hemolymph volume. Samples were cleared by centrifugation at 4°C and assayed for glucose concentrations using the HK or GO glucose assay kit (Sigma-Aldrich) according to the manufacturer’s instructions.

### 7.2. Experiment 2: long-term ap-AKH injection study

A repeated ap-AKH-injection study was carried out to investigate long-term effects. *A. californica* (n = 5/group) were first weighed to establish baseline body mass, then injected with 15 µg of ap-AKH or ASW vehicle every 48 hours for 20 consecutive days. On injection days, *A. californica* were gently removed from their cages, injected, returned to their cages, and given 20 g Romaine lettuce. *A. californica* were then left unperturbed for 3 hours. At that time, any remaining lettuce was blotted dry, weighed to estimate food consumption, and discarded. Injections were omitted on alternating days, but animals were otherwise fed and weighed in the same manner. All *A. californica* were sacrificed on Day 20, 24 hours after the final injection. Immediately before sacrifice, a terminal hemolymph sample was taken for glucose assay as described in Experiment 1. At sacrifice, hepatopancreas (HP) samples were quickly dissected, snap frozen on dry ice, and stored at −70°C until glycogen analysis. To assess the impact of ap-AKH treatment on the gonad, the OVT was removed, weighed for each animal, and immersion-fixed in Bouin’s fixative for 48 hours. OVT were then transferred to 70% ethanol until the histological assessment of oocyte diameters.

Terminal hemolymph glucose was assayed as described in Experiment 1. HP glycogen content was measured using the phenol-sulfuric acid method described previously [39]. Samples were assayed in triplicates and normalized for HP wet mass.

To assess oocyte maturation, samples of OVT from *A. californica* were dehydrated, cleared, and embedded in paraffin. All OVT samples were taken from the area closest to the confluence of the small hermaphroditic duct. Once embedded, OVT samples were sectioned at 12-µm thickness, mounted on gelatin-subbed slides, stained with hematoxylin and eosin (H&E), dehydrated, and coverslipped. Oocyte diameter measurements were performed on coded slides by an observer blind to their identity. For each animal, oocytes were scored from 4 non-adjacent sections separated by ∼90 µm (diameter reported for the largest oocytes [40]). This ensured that each oocyte was not scored multiple times. Because oocytes are often elliptical, each oocyte was measured at both its widest diameter and narrowest diameter, and these values averaged to obtain the mean diameter for that oocyte. A total of 60 oocytes from 3 randomly chosen areas were scored per section using a calibrated ocular micrometer. Thus, mean oocyte diameter for each animal reflects the average of 240 oocytes.

### 7.3. Experiment 3: acute effect of ap-AKH on excretion of feces

During the course of our long-term injection study, we noted the appearance of fecal matter within minutes of ap-AKH injection in *A. californica*. To quantify this phenomenon, we collected feces from the cages 1 hr after the injection of 15 µg ap-AKH or ASW (n = 7/group). Fecal pellets were air-dried then weighed.

### 7.4. Experiment 4: acute effect of ap-AKH on body mass

To elucidate the time course of acute body mass change without the confounding effect of feeding, *A. californica* that had been food-deprived for 24 hours were injected with either 15 µg of ap-AKH or ASW vehicle (n = 5/group). Body mass was recorded for each individual immediately before injection and at 10 min, 30 min, and 1, 2, 3, 4, 6, and 24 hours after injection. No feeding occurred during the experiment.

### 7.5. Experiment 5: differential *in vivo* effects of ap-AKH and ap-GnRH

A 3-day study was conducted to compare the effects of ap-AKH and ap-GnRH on parapodial opening (Day 1), body mass (Day 1), and feeding (Day 3) *in vivo*. On Day 1 and 3, *A. californica* (n = 5/group) were injected with 15 µg of ap-AKH, 15 µg ap-GnRH, or ASW vehicle. Synthetic ap-GnRH (pQNYHFSNGWYA-amide) was generated and prepared as previously described [27].

On Day 1 and 24 hours after the last feeding, body mass was recorded prior to and at 2, 4, 6, and 24 hours after injection. Parapodial opening, a previously established motor effect of ap-GnRH [27], was measured at 5 and 10 minutes post-injection as described [27]. No feeding occurred on Day 1. On Day 2, animals were fed and allowed to recuperate. On Day 3, 20 g Romaine lettuce was provided following injections, and remaining lettuce was blotted and weighed at 1 and 3 hours post-injection.

### 8. Statistical analyses

Two-way repeated measures ANOVA followed by Bonferroni’s multiple comparisons post hoc test were used to analyze the repeated measurements of hemolymph glucose (Experiment 1), body mass (Experiments 2, 4, 5), and food consumption (Experiments 2 and 5). In cases when data were normalized to baseline levels, the baseline value (100%) was excluded from statistical analysis due to the lack of variance. Mann-Whitney U-test was used to analyze single-time point data on OVT mass, oocyte diameter, HP glycogen stores, terminal hemolymph glucose, fecal pellet mass, and total food consumed in Experiments 2, 3, and 5. During the course of Experiment 2, one ASW-injected control animal displayed sickly behavior (immobility, excessive inking, skin lesion) 48 hours after the commencement of experiment and was excluded from final analyses. Statistical analyses were carried out using Prism 6 (GraphPad, San Diego, CA). Differences were considered significant when p<0.05.

## Results

### 1. Cloning of ap-AKH

**Figure 1:**
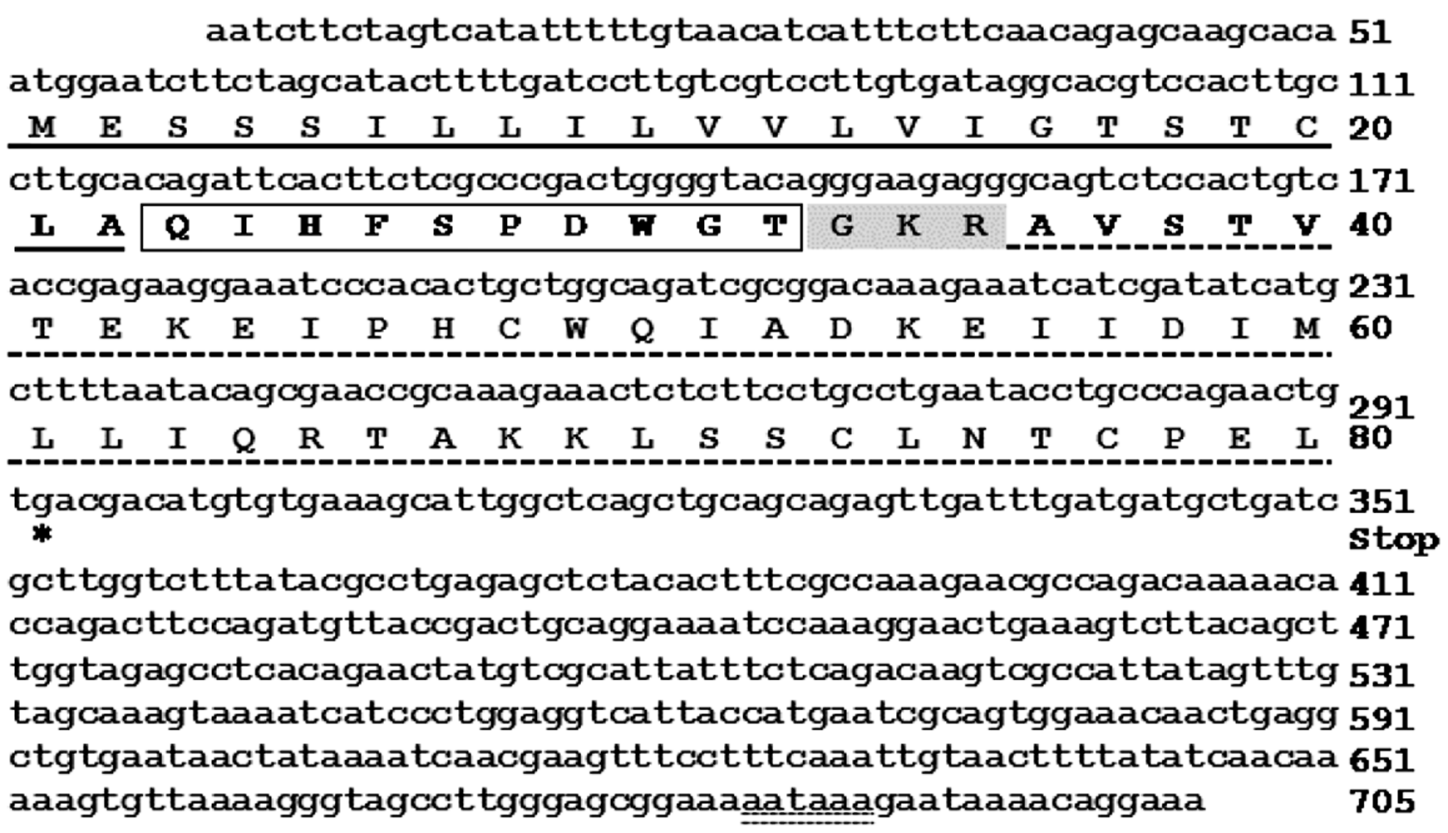
Nucleotide and deduced amino acid sequences of prepro ap-AKH. Full-length nucleotide sequence (lower case letters) and deduced amino acid sequence (upper case letters) of prepro ap-AKH (GenBank Accession #JQ929303.2). The signal peptide is underlined with a solid line; the mature ap-AKH decapeptide is boxed, and the associated peptide is underlined with a dashed line. The dibasic cleavage site and α-amidation signal are highlighted in grey. The asterisk denotes the stop codon and the polyadenylation site is underlined with double dotted lines.

The cloned prepro *ap-AKH* spans 705 bp and includes a 51-bp 5′ UTR, a 240-bp open reading frame that encodes a putative preprohormone of 80 amino acids, and a 414-bp 3′ UTR (Figure 1). The deduced prepro ap-AKH consists of a 22-amino acid signal peptide, a 10-amino acid ap-AKH, a dibasic processing site with an α-amidation signal (GKR), and a 45-amino acid ap-AKH-associated peptide (Figure 1; GenBank Accession# JQ929303.2). The deduced amino acid sequence of ap-AKH is pQIHFSPDWGT-amide (Figure S1). ap-AKH shares several highly conserved amino acids with other protostomian AKH and CRZ, including Glu^1^ and Trp^8^ that are universal to all members of the AKH and CRZ families (Figure S1).

### 2. RT-PCR

**Figure 2:**
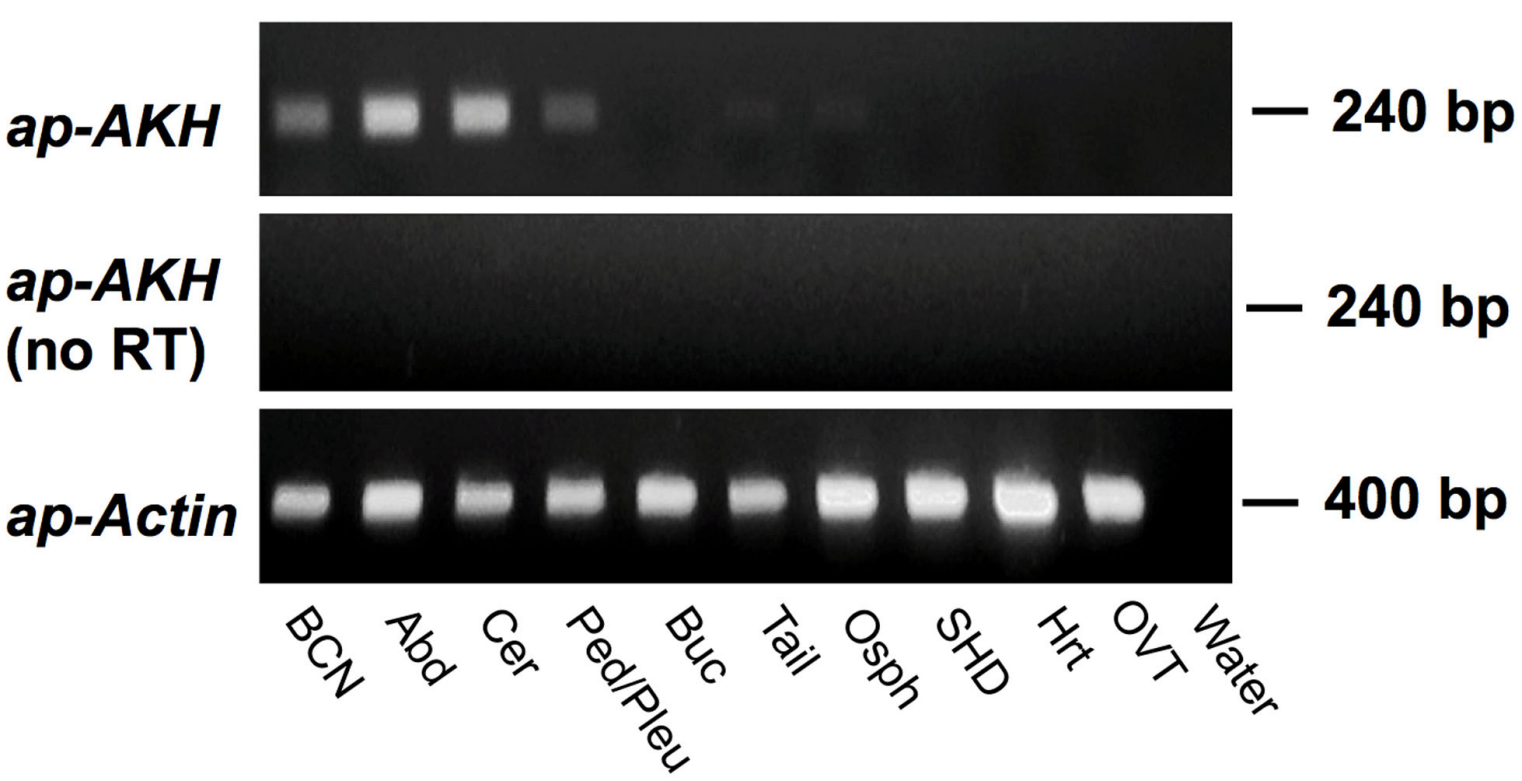
RT-PCR of *ap-AKH* in central and peripheral tissues of *A. californica*. *ap-AKH* is expressed in the CNS but not peripheral tissues of *A. californica* (top panel). Negative control (middle panel) was RNA with no RT, and positive control (bottom panel) used *ap-Actin* primers. Abbreviations: BCN, bag cell neurons; Abd, abdominal ganglion; Cer, cerebral ganglia; Ped/Pleu, pedal/pleural ganglia; Buc, buccal ganglia; Tail, tail muscle; Osph, osphradium; SHD, small hermaphroditic duct; Hrt, heart; OVT, ovotestis; Water, no template.

RT-PCR revealed that the *ap-AKH* is expressed in the BCN, abdominal ganglion, cerebral ganglion and pedal/pleural ganglia (Figure 2). Negative controls without RT or lacking a template did not yield any product (Figure 2).

### 3. Localization of ap-AKH-ir in the CNS of *A. californica*

**Figure 3:**
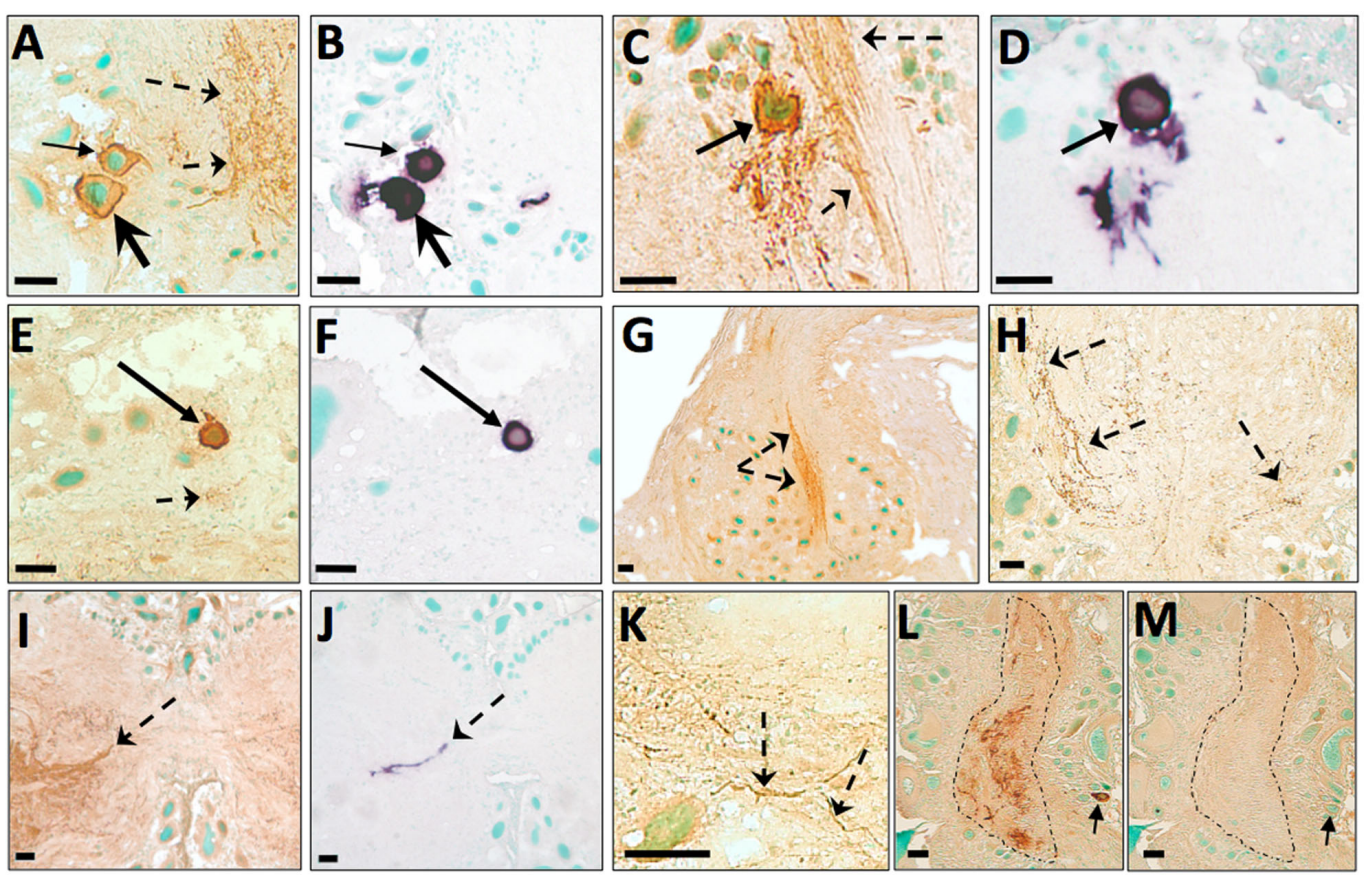
ISH and ICC of *A. californica* CNS. Representative photomicrographs of ISH (purple; B, D, F, J) or ICC (brown; A, C, E, G, H, I, K, L, M) staining in the CNS of *A. californica*, including abdominal (A, B, L, M), buccal (K), cerebral (C, D, I, J), pleural (E, F), and pedal (H) ganglia, and bag cell neurons (G). Preadsorption with ap-AKH (M) completely abolished ap-AKH-ir signal compared to an adjacent section (L). Green = methyl green nuclear counterstain. Solid arrows denote ap-AKH-ir or ap-AKH transcript-positive neuronal cell bodies; arrow pairs of the same shape in panels A/B, C/D, E/F, and L/M point to identical neurons in adjacent sections. Dashed arrows in panels A, C, E, G, H, I, and K point to ap-AKH-ir fibers in the neuropil regions of ganglia. Dashed arrow in panel J points to *ap-AKH* transcript-positive fibers. Dashed outline in panel L surrounds ap-AKH-ir fibers in the neuropil region of the abdominal ganglia, and in M surrounds the same region of neuropil devoid of signals. Scale bars = 50 µm.

**Figure 4:**
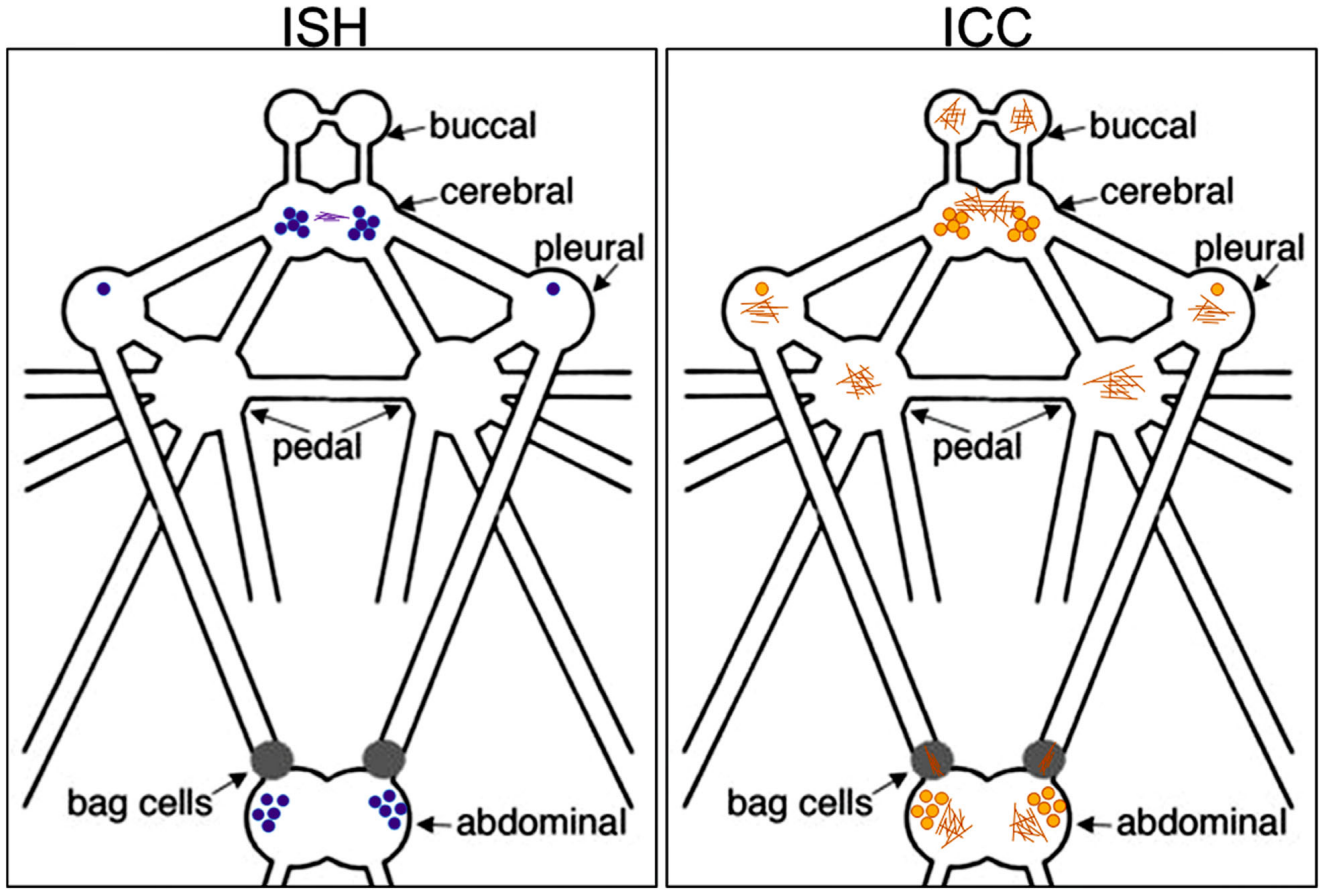
Diagrammatic representation of *ap-AKH* transcript and peptide in *A. californica* CNS. The relative abundance and location of neurons and fibers positive for *ap-AKH* transcript (purple dots and lines; left) or ap-AKH peptide (tan dots and lines; right) are shown.

ICC using a specific anti-ap-AKH antiserum revealed ap-AKH-ir neurons in the lateral portions of the abdominal ganglion (Figure 3A), the dorsal-medial portion of each cerebral hemiganglion (Figure 3C), and the anterior-medial portion of the pleural ganglia (Figure 3E). ap-AKH-ir neurons were found in greatest abundance in the abdominal and cerebral ganglia, with each ganglia containing 5–7 neurons (Figure 4); the pleural ganglia had fewer ap-AKH-ir neurons, with only 1–2 neurons per ganglia (Figure 4). Dense ap-AKH-ir fibers were also found in all central ganglia, indicating transport of the peptide throughout the CNS (Figures 3A, C, E, G, H, I, K; dashed arrows). Pedal (Figure 3H) and buccal (Figure 3K) ganglia were devoid of ap-AKH-ir neuronal cell bodies, but each possessed abundant ap-AKH-ir fibers in the neuropil region. The BCN were also devoid of ap-AKH-ir neuronal cell bodies but contained robust ap-AKH-ir fibers (Figure 3G). The specificity of our antiserum was confirmed when preadsorption with 20 µg/ml ap-AKH abolished all staining (Figures 3L, M).

### 4. Localization of ap-AKH mRNA in the CNS of *A. californica*

ISH using sections adjacent to those used for ICC revealed an excellent match between the presence of ap-AKH transcript and peptide (Figure 4). Our ISH revealed the highest abundance of positive neurons in the abdominal and cerebral ganglia, followed by the pleural ganglia (Figures 3B, D, F). Again, buccal, pedal, and the BCN were devoid of any signal (Figure 4). Interestingly, we observed sparse but clear ISH signal near the commissural bundle between the two cerebral hemiganglia (Figure 3J). This was co-localized with ap-AKH-ir fibers in adjacent ICC sections (Figure 3I), suggesting ap-AKH translation may occur outside the neuronal cell bodies.

### 5. Experiment 1: acute effects of ap-AKH on hemolymph glucose levels

**Figure 5:**
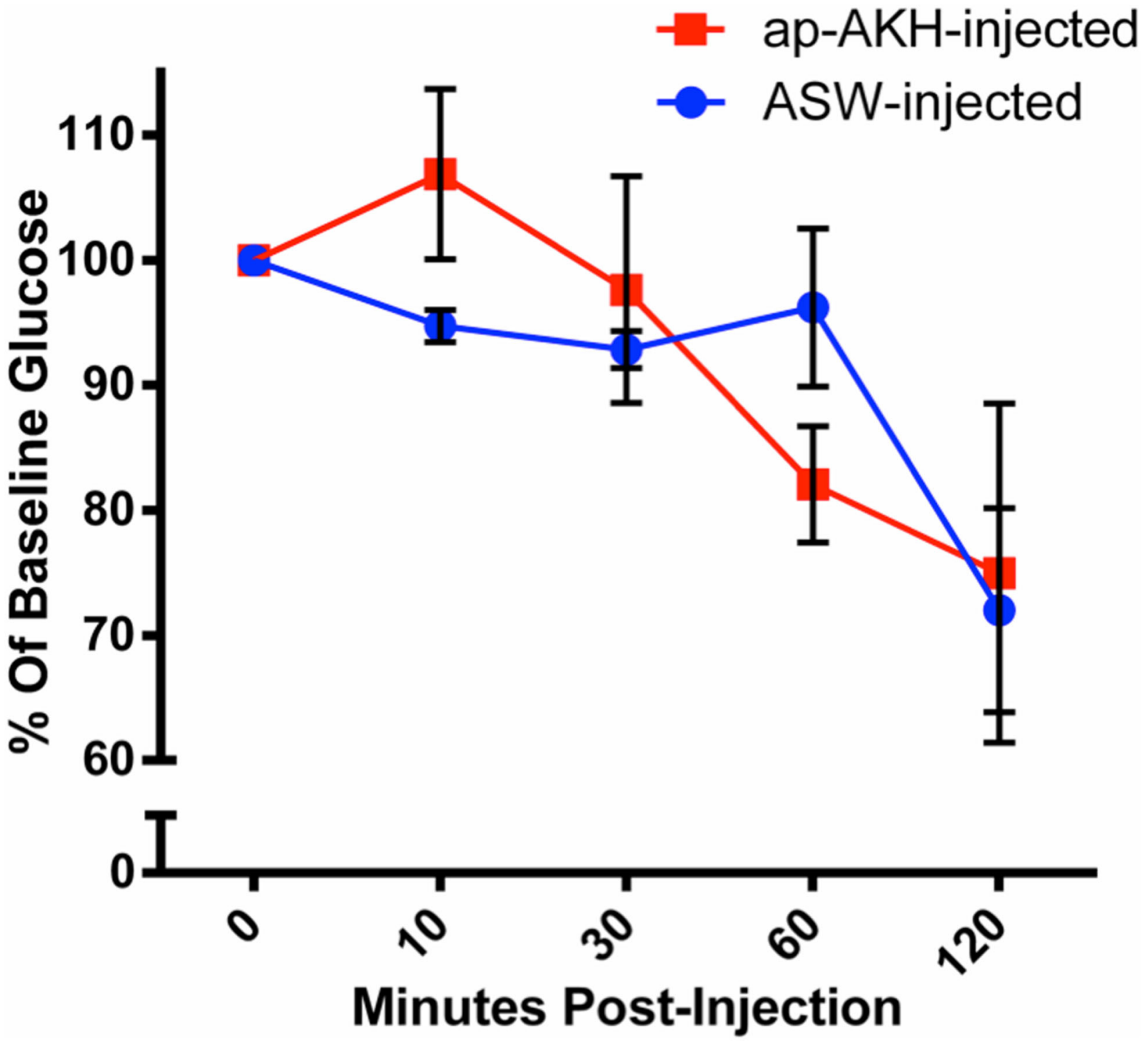
Hemolymph glucose was not acutely altered by a single ap-AKH injection. Hemolymph glucose was normalized against baseline levels at Time 0. ASW or ap-AKH was injected immediately after Time 0. No significant differences were detected between ASW-injected and ap-AKH-injected animals. Data are mean ± SEM; n = 5/group.

A single injection of 30 µg ap-AKH had no significant effect [F(1, 8) = 0.03, p = 0.86] on circulating glucose levels at the time points examined (Figure 5). There was a reduction in hemolymph glucose over the course of the experiment [F(4, 32) = 8.456, p<0.0001] in both groups (Figure 5). This decrease was typical for repeated hemolymph sampling [32], [37].

### 6. Experiment 2: long-term ap-AKH injection study

**Figure 6:**
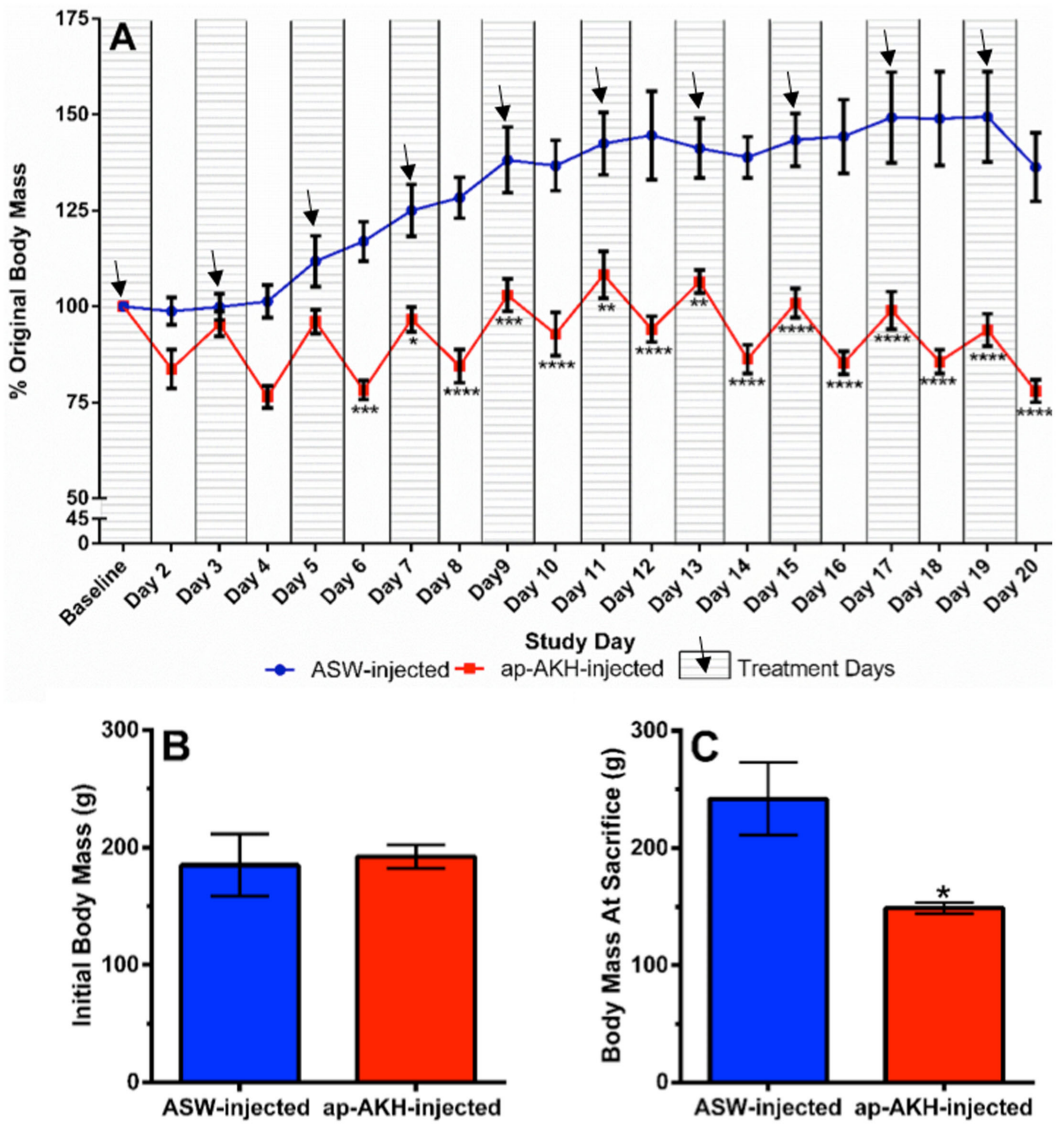
Long-term effects of ap-AKH injections on body mass. A) Time course of body mass change in response to ap-AKH injections over time. Daily body mass was presented as a percentage of baseline body mass. Hatched zones with arrows indicate injection days, and white zones indicate non-injection days. B) Initial body mass was not different between ASW- and AKH-injected groups. C) Terminal body mass was significantly reduced in ap-AKH-injected group. * = p<0.05, ** = p<0.01, *** = p<0.001, and **** = p<0.0001 compared to ASW-injected group. Data are mean ± SEM; n = 4–5/group.

Initially, baseline body mass was not different between ap-AKH and ASW-injected groups (Figure 6B), thus all data in Figure 6A were presented as percent of the baseline. One control animal was excluded from data analysis due to poor health (see Materials and Methods, Section 8). Two-way repeated measures ANOVA showed significant effects of treatment [F(1, 7) = 30.89, p = 0.0009], time [F(18, 126) = 25.05, p<0.0001], and treatment×time interaction [F(18, 126) = 12.96, p<0.0001] on body mass. Bonferroni’s post hoc test revealed significantly reduced body mass in ap-AKH-injected animals compared to controls from Day 6 onward (Figure 6A). Interestingly, body mass of ap-AKH-injected animals consistently declined to ∼75% of their baseline mass 24 hours post-injection, but recovered to ∼100% baseline after another 24 hours (Figure 6A). At sacrifice, the body mass of ap-AKH-injected group was significantly decreased compared to ASW-injected group (p = 0.015; Figure 6C).

**Figure 7:**
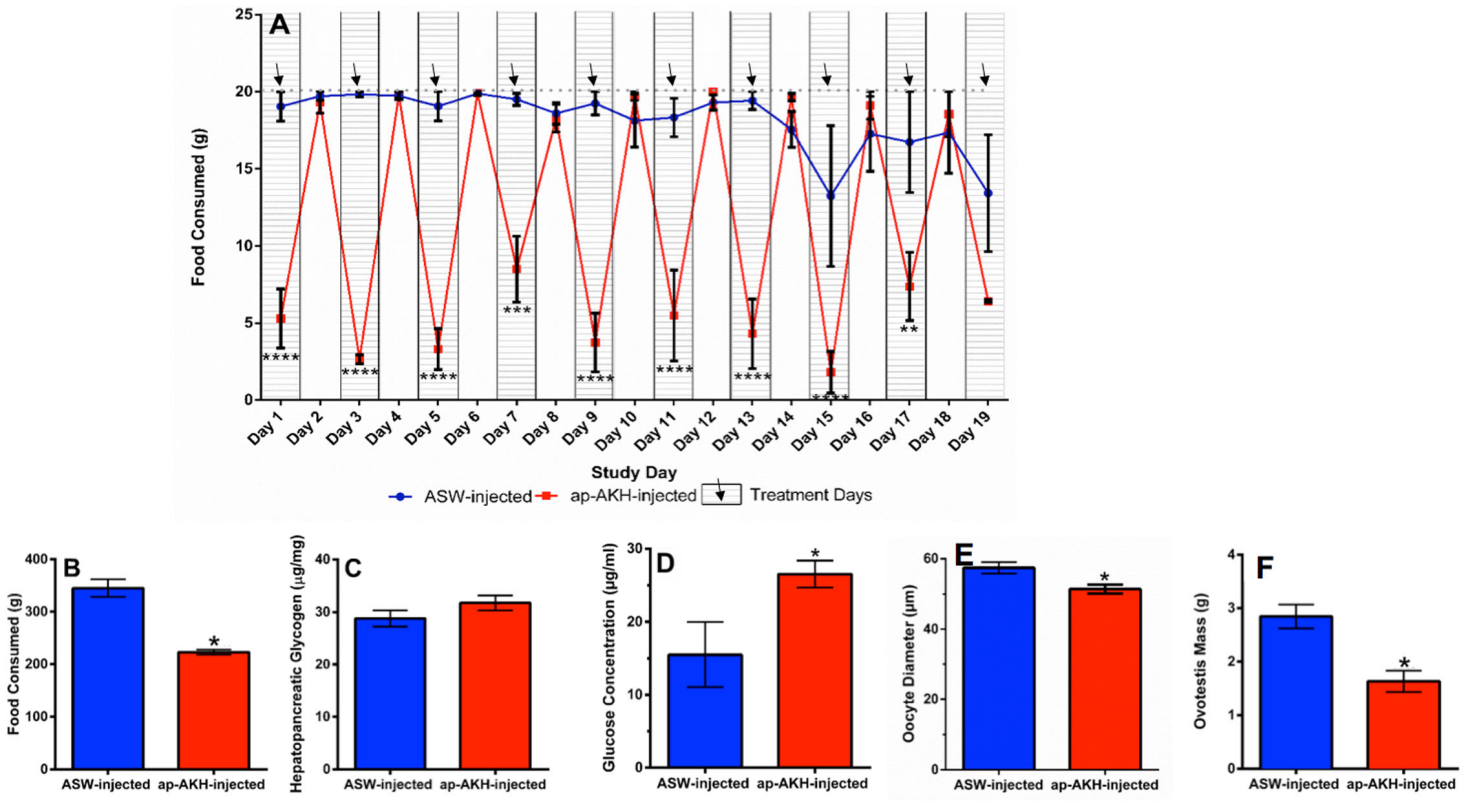
Long-term effects of ap-AKH injections on feeding, metabolic parameters, and OVT. A) Time course of food consumption in response to ap-AKH injections. Hatched zones with arrows indicate injection days, and white zones indicate non-injection days. The dotted line at 20 g indicates maximum food consumption possible per day. B) Total food consumption over the 20-day experimental period was significantly reduced in ap-AKH injected animals. C) HP glycogen stores at the end of the experiment were not altered by ap-AKH. D) Hemolymph glucose levels were higher at sacrifice in the ap-AKH-injected group. E) Mean oocyte diameter and F) OVT mass at sacrifice were reduced in the ap-AKH-injected group. * = p<0.05, ** = p<0.01, *** = p<0.001, and ****p<0.0001 compared to ASW-injected group. Data are mean ± SEM; n = 4–5/group.

The role of AKH in insect metabolism [4], [41], [42] suggested ap-AKH may regulate diverse metabolic parameters in *A. californica*. Guided by this information, we first assessed food consumption as a general gauge of metabolism. Two-way repeated measures ANOVA revealed significant effects of treatment [F(1, 7) = 60.94, p = 0.0001], time [F(18, 126) = 13.74, p<0.0001] and treatment×time interaction [F(18, 126) = 10.97, p<0.0001] on food consumption. Bonferroni’s post hoc test revealed that food consumption was consistently reduced in ap-AKH-injected animals for at least the first 3 hours post-injection (the duration of time that food was present), but restored to normal on non-injection days (Figure 7A). Overall, ap-AKH-injected animals consumed significantly less food in total over the course of the study (p = 0.015; Figure 7B). Despite the decreased food consumption, terminal HP glycogen stores in the ap-AKH-injected group were not significantly reduced compared to the control group (Figure 7C). Interestingly, circulating glucose was elevated at the time of sacrifice (24 hours after the final ap-AKH-injection; p = 0.015; Figure 7D).

Mean oocyte diameter was modestly but significantly reduced after repeated ap-AKH injections over 20 days compared to controls (p = 0.030; Figure 7E). A significant reduction in wet OVT mass was also observed in ap-AKH-injected animals (p = 0.015; Figure 7F).

### 7. Experiment 3: acute effect of ap-AKH on excretion of feces

**Figure 8:**
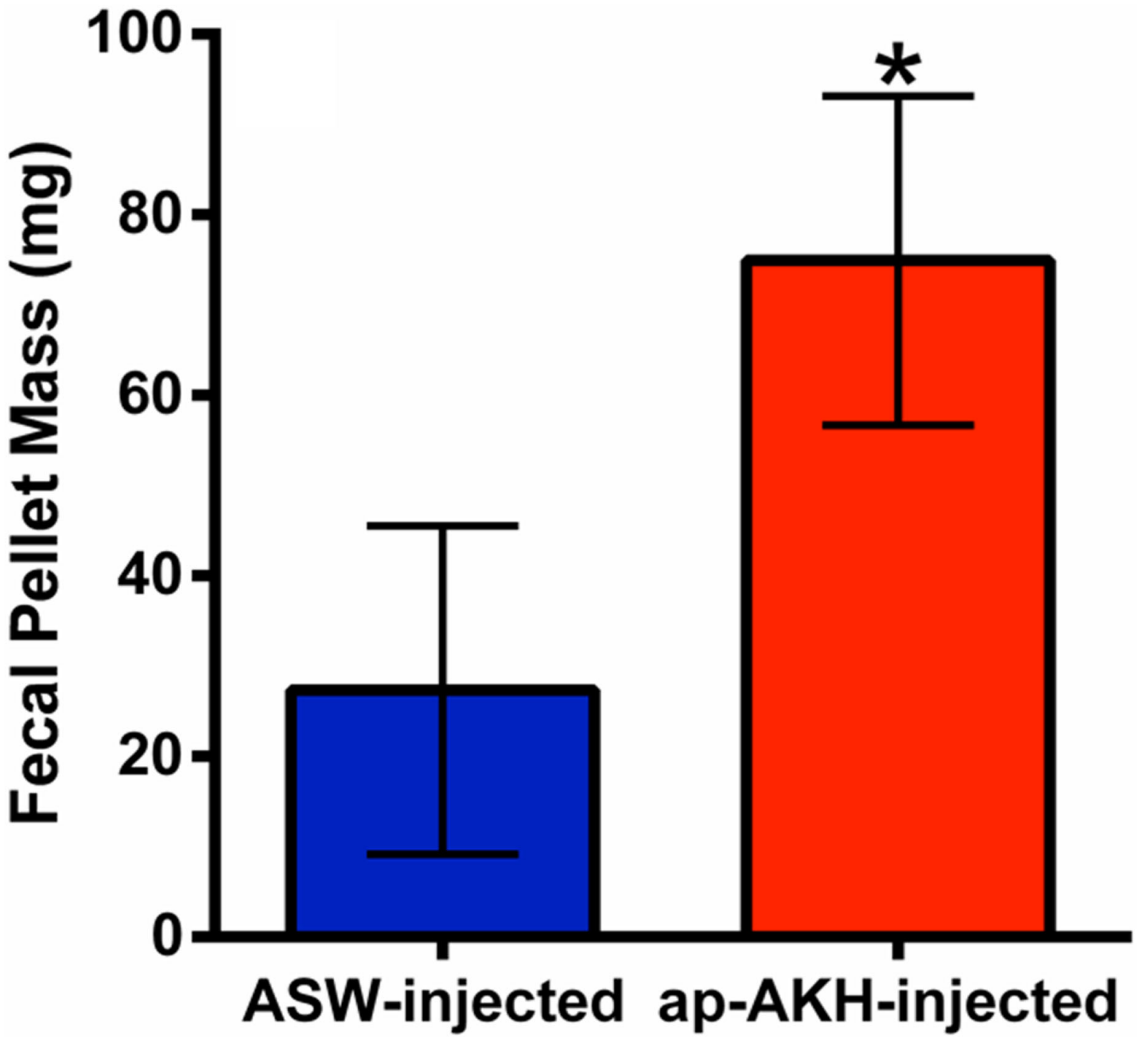
Acute effect of ap-AKH on excretion of feces. ap-AKH injection significantly increases fecal pellet mass within one hour of injection. * = p<0.05 compared to ASW-injected group. Data are mean ± SEM; n = 7/group.

At 1 hour post-injection, ap-AKH significantly increased the excretion of feces compared to control animals (p = 0.026; Figure 8), suggesting the stimulation of gut motility.

### 8. Experiment 4: acute effect of ap-AKH on body mass

**Figure 9:**
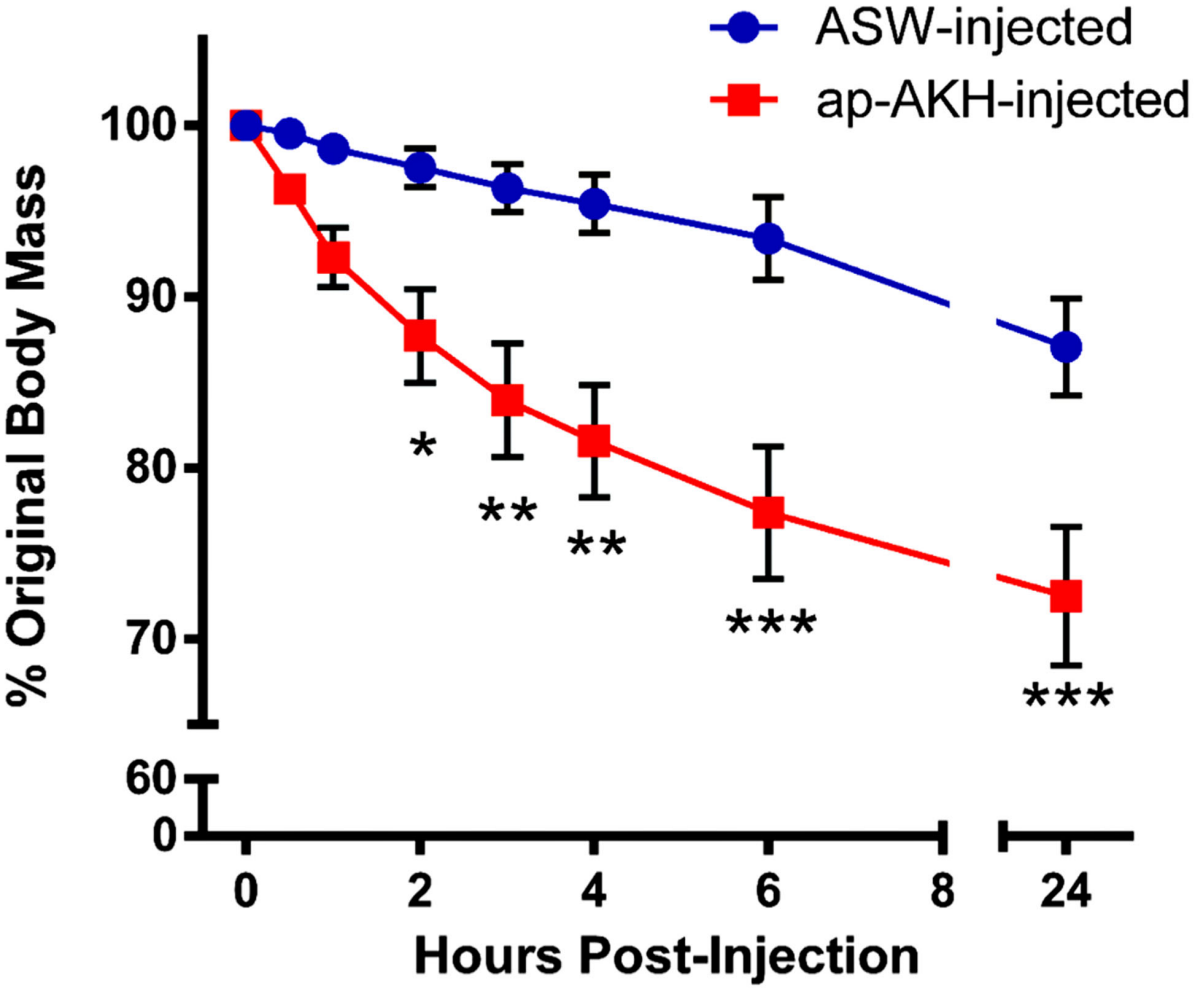
A single injection of ap-AKH induced rapid and sustained loss of body mass. Significant reductions in body mass were found at 2, 3, 4, 6, and 24 hours in ap-AKH-injected group. * = p<0.05, ** = p<0.01, and *** = p<0.001 compared to ASW-injected group. Data are mean ± SEM; n = 5/group.

Since ap-AKH reduced body mass at 24 hours post-injection in Experiment 2 (Figure 6A), we examined the detailed time course of this change without the confounding effects of feeding. Two-way repeated measures ANOVA indicated significant effects of treatment [F(1, 8) = 13.02 p = 0.0069], time [F(6, 48) = 42.62, p<0.0001], and treatment×time interaction on food consumption [F(6, 48) = 6.132, p<0.0001]. Bonferroni’s post hoc test indicated that, even in the absence of feeding, ap-AKH-injection significantly reduced body mass between 2 and 24 hours post-injection (Figure 9).

### 9. Experiment 5: differential *in vivo* effects of ap-AKH and ap-GnRH

**Figure 10:**
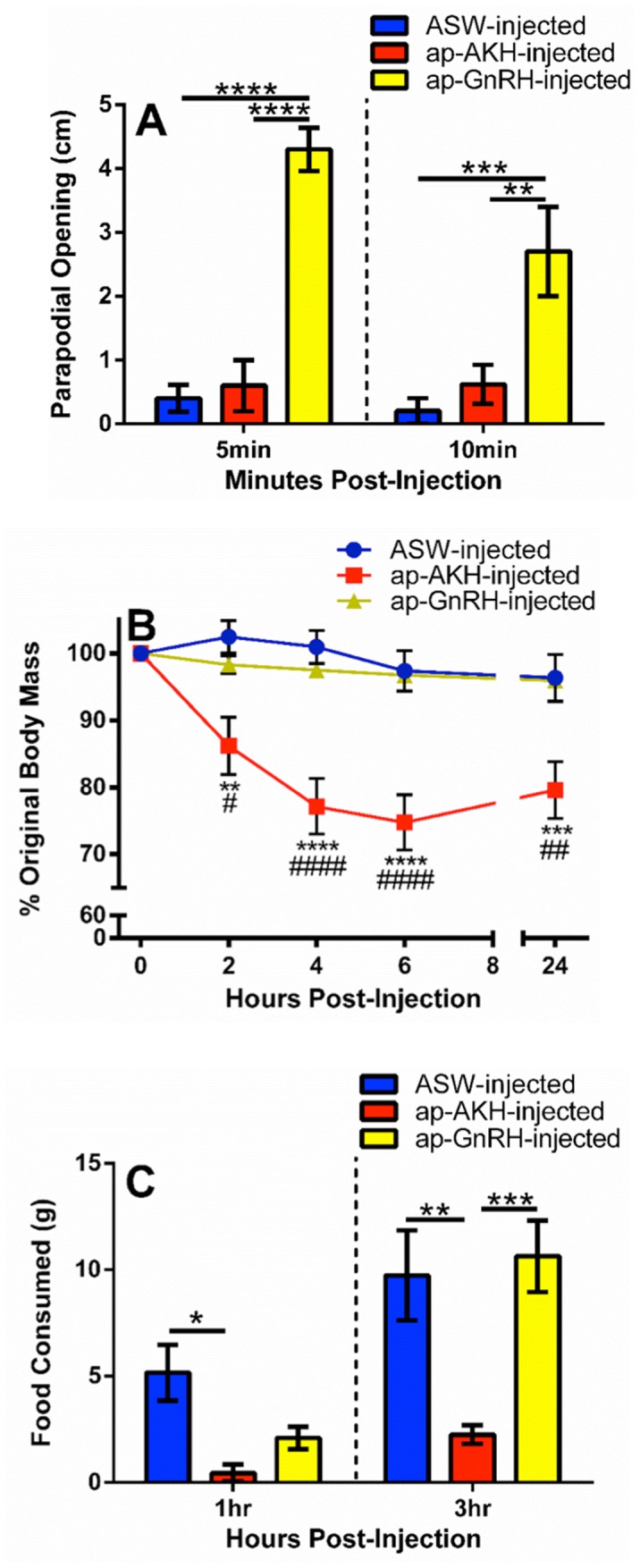
Comparisons of *in vivo* effects of ap-AKH and ap-GnRH revealed no overlap. A) A single injection of ap-GnRH, but not ap-AKH, stimulated parapodial opening at 5 and 10 minutes post-injection. B) A single injection of ap-AKH, but not ap-GnRH, reduced body mass between 2 and 24 hours post-injection. C) A single injection of ap-AKH, but not ap-GnRH, suppressed food intake at 1 and 3 hours post-injection. * = p<0.05, ** = p<0.01, *** = p<0.001, ****p<0.0001. In panel B, # denotes differences between ap-AKH and ap-GnRH groups while *denotes differences between ap-AKH and ASW groups. Data are mean ± SEM; n = 5/group.

ap-AKH and ap-GnRH are thought to share a common ancestor [2]. To differentiate the function of these two homologs, we compared several *in vivo* effects of ap-AKH and ap-GnRH, including parapodial opening, body mass change, and feeding. Two-way repeated measures ANOVA revealed a significant effect of treatment [F(2, 12) = 30.7, p<0.0001] on parapodial opening. Bonferroni’s post hoc test showed that ap-GnRH stimulated significant parapodial opening at 5 and 10 minutes post-injection, but ap-AKH had no effect compared to ASW at either time (Figure 10A). There were significant effects of treatment [F(2, 12) = 14.49, p = 0.0006], time [F(3, 36) = 18.31, p<0.0001], and treatment×time interaction [F(6, 36) = 5.597, p = 0.0003] on body mass. Bonferroni’s post hoc test revealed ap-AKH injection significantly reduced body mass of *A. californica* at 2, 4, 6, and 24 hours post-injection, but ap-GnRH had no effect at any time (Figure 10B). Lastly, analysis of food consumption revealed significant effects of treatment [F(2, 12) = 7.93, p = 0.0064], time [F(1,12) = 69.64, p<0.0001], and treatment×time interaction [F(2, 12) = 10.74, p = 0.0021]. Bonferroni’s post hoc test revealed ap-AKH significantly reduced food consumption at 1 and 3 hours post-injection, but, again, ap-GnRH had no effect at either time (Figure 10C).

## Discussion

This study is the first to report the cloning, localization, and functional characterization of a molluscan AKH. Results from RT-PCR, ISH, and ICC show that ap-AKH is synthesized in specific neurons of the cerebral, abdominal, and pleural ganglia, and that the mature peptide is transported throughout the CNS (Figures 2, 3, 4). *In vivo* functional characterization reveals that ap-AKH suppresses feeding (Figures 7A, B), reduces body mass (Figure 9), and alters gut function (Figure 8), but does not activate reproduction (Figures 7E, F). In fact, ap-AKH exhibits little overlap with the function and expression pattern of *ap-GnRH* [27–29; this study], suggesting these two homologous peptides have diverged functionally over the course of evolution.

Our cloning results confirm the reported ap-AKH sequence [2] and reveal that it shares several features common to AKH peptides in diverse taxa (Figure S1). For example, the deduced mature ap-AKH peptide contains several highly conserved amino acids (residues 1, 4, 6, 8; Figure S1) and retains important motifs conserved within the GnRH superfamily [2], including the potential N-terminal pyroglutamylation and the presumed C-terminal amidation (Figure 1 and Figure S1).

RT-PCR results (Figure 2) suggest that *ap-AKH* is expressed exclusively within the CNS. ISH confirms CNS expression by demonstrating robust signals in defined neurons of abdominal, cerebral, and pleural ganglia (Figures 3, 4). ICC of adjacent sections shows that neuronal cell bodies positive for the transcript are also positive for the peptide, supporting *de novo* ap-AKH synthesis by these neurons. A notable discrepancy between RT-PCR and ISH is that ISH does not detect *ap-AKH* in the BCN, two clusters of neuroendocrine neurons that secrete an ovulation hormone called egg-laying hormone (ELH). This could be due to low levels of *ap-AKH* expression in the BCN that escape detection by the less sensitive ISH. The absence of ISH signal in BCN is also consistent with the absence of ICC signal in cell bodies of the BCN (Figure 3G). Interestingly, ICC detects robust fiber staining in all ganglia, suggesting ap-AKH is transported throughout the CNS. For example, although pedal and buccal ganglia do not contain cell bodies with positive ISH or ICC signals, they are richly innervated with ap-AKH-ir fibers (Figures 3H, K, 4).

ap-AKH-ir neurons in the cerebral ganglia are located near the dorsal-medial surface of the ganglia. The location and size of these ap-AKH-ir neurons are highly consistent with those of neurons in the cerebral C clusters. The C clusters are two symmetrical clusters each with 80 neurons and well-characterized connectivity and morphological properties [43]. This suggests some ap-AKH-ir neurons may be a subset of the C clusters or may locally modulate the C cluster neurons. Interestingly, several C cluster neurons, including the cerebral-abdominal inter-ganglionic (CAI) neurons [44], send projections through the pleurovisceral connective (PVC) nerves, which are the nerves that connect the abdominal and head ganglia. Moreover, CAI neurons have axon terminals in the abdominal, pleural, and pedal ganglia, and coordinate visceral actions of the abdominal ganglion with sensory input and motor programs [44]. It is possible that some of the ap-AKH-ir fibers that traverse through the BCN clusters come from the cerebral C clusters via the PVC nerves (Figure 3G). Collectively, these observations raise the possibility that ap-AKH may act as an inter-ganglionic signal between the head ganglia and abdominal ganglion to coordinate visceral, motor, sensory and perhaps reproductive processes [43]–[46].

Consistent with the relative abundance of ap-AKH-positive neurons in the cerebral and abdominal ganglia, many of the acute *in vivo* responses induced by ap-AKH involve efferent targets of the abdominal and cerebral ganglia. For example, injection of ap-AKH inhibited feeding (Figure 7A) and increased the excretion of feces (Figure 8); the latter may result from enhanced gut contraction. The abdominal ganglion has well-established innervations to the rectum via the genital nerve, and can induce contractions in the gut. Further, CAI neuron activity simultaneously modulates feeding behavior and genital nerve activity [44]. Together with the neuroanatomical overlap between ap-AKH-ir and the C cluster neurons, these data support the notion that ap-AKH may mediate the regulatory actions of cerebral and/or abdominal ganglion on feeding and gut function, thereby coordinating food intake, digestion, and excretion.

Loss of roughly 25% body mass was also acutely induced by ap-AKH (Figures 9, 10B). The acute nature of this effect suggests water loss as the most likely cause and implicates ap-AKH in volume regulation. Supporting this, the hemolymph of *A. californica* makes up about 75% of their body mass and represents a physiological substrate that can be dynamically regulated [47]. *Aplysia* species are known to volume regulate, probably through neuroendocrine signals [48], in response to osmotic changes in the environment [49], [50]. *A*. *californica* can monitor the tonicity of the environment with osphradium [51]–[53], a chemo- and osmosensory organ, and may control water and ion trafficking through several outlets including the skin [54], digestive tract [49], and renal system [55], [56]. Like feeding and gut motility, water trafficking may be controlled, in part, by the abdominal ganglion [45], [52], [57], [58]. Moreover, some neurons in the abdominal ganglion associated with volume regulation are modulated by the CAI neurons in the cerebral ganglia [44]. Thus, this particular *in vivo* effect remains consistent with the anatomical localization of ap-AKH. To our knowledge, volume regulation by AKH has never been described in other phyla and may represent a unique specialization of molluscan AKH.

In insects, AKH is a neurohormone that mobilizes energy reserves (i.e. diacylglycerol or trehalose) during times of high energy expenditure [7]. Since HP glycogen and hemolymph glucose are the primary forms of stored and circulating carbohydrates, respectively, in *A. californica*, we examined the effects of ap-AKH on these two parameters [32], [37], [38]. ap-AKH did not acutely alter circulating glucose (Figure 5), nor did it affect HP glycogen after repeated injections (Figure 7C). Interestingly, the ap-AKH-injected group had elevated circulating glucose at the end of the 20-day study (Figure 7D). These findings suggest that ap-AKH is not an acute, but instead a slow-acting, hyperglycemic factor. Alternatively, the elevated terminal circulating glucose may have resulted secondarily from dehydration, as suggested by the lower body mass in ap-AKH-treated animals (Figures 6A, C). The observation that HP glycogen stores were unaffected was unexpected since ap-AKH-injected *A. californica* had lowered food intake over the course of the 20-day study (Figure 7B) and potentially lower efficiency of digestion due to increased gut motility (Figure 8) [59]. We posit that the maintenance of glycogen stores despite reduced food intake and elevated circulating glucose may result from behavioral or metabolic adaptations [59]–[61] yet to be elucidated. It is interesting to note that long-term ap-AKH injections suppressed weight gain (Figure 6C) and at the same time reduced oocyte maturity and OVT mass (Figures 7E, F), suggesting a reallocation of energetic reserves to favor energy conservation similar to changes observed seasonally in other mollusks [60], [62], [63]. Lastly, although long-term ap-AKH treatment elevated terminal hemolymph glucose, it is currently unclear if ap-AKH regulates metabolism directly or indirectly by modifying food intake and gut motility.

After 20 days, ap-AKH-treated *A. californica* had significantly reduced mean oocyte diameter (Figure 7E) and OVT mass (Figure 7F), suggesting oogenesis was inhibited. The number of oocyte number has not been quantified and remains a possible effect of ap-AKH. In general, ap-AKH appears to suppress gonadal function. Of interest, the long-term reproductive effects of ap-AKH resemble the temperature-driven diapause-like state characterized by reproductive dormancy [64] and lower body mass [40] in *A. californica* during winter and spring.

The simultaneous presence of ap-AKH and ap-GnRH in *A. californica* provides a unique opportunity to investigate the functional divergence of two homologous neuropeptides within a single species [28]. Previous data suggest that ap-GnRH is involved in the motor and behavioral control of *A. californica* [27]. We measured three parameters known to be affected by ap-AKH (current study) or ap-GnRH [27] and found no functional overlap between these two neuropeptides. Specifically, only ap-AKH decreased feeding and body mass, and only ap-GnRH enhanced parapodial opening (Figure 10). Of interest, although ap-GnRH reduced feeding in the previous study [27], it was ineffective in the current study (Figure 10C). This was likely due to the current experimental design of taking the first feeding measurement at 1 hour post-injection (see Materials and Methods, Section 7.5), a time point beyond the effectiveness of ap-GnRH [27]. It appears that both ap-GnRH and ap-AKH can acutely suppress feeding, but the effect of ap-AKH lasts much longer than ap-GnRH. Interestingly, anorexic effect is thought to be an ancient function of the GnRH family peptides [18]–[20], [27].

In sum, neuroanatomical distribution and functional results indicate that ap-AKH is produced primarily in the abdominal and cerebral ganglia where it may be involved in the coordination of visceral and metabolic processes. ap-AKH induces both acute and long-term effects. The former include the acute reduction of body mass and feeding; the latter include reduced OVT mass and oocyte diameter, suppressed long-term body mass gain, and elevated terminal hemolymph glucose, which may be a direct metabolic effect or an effect secondary to dehydration. Together, these results broadly suggest that ap-AKH modulates metabolism, volume regulation, feeding, and gut motility. Further, the biological activities of ap-AKH and ap-GnRH exhibit little overlap. Our results raise some interesting possibilities regarding the functional roles of these peptides other than reproductive activation. Further, they lay the groundwork for future investigations on the functions of the GnRH superfamily members in diverse protostomian taxa.

## Supporting Information

Figure S1 **Alignment of amino acid sequences of AKH and Crz.** Amino acid sequences of ap-AKH (*Aplysia californica*; AFN66119.1) aligned with: AKH Limpet (*Lottia gigantea*; AMQO01003719.1), AKH I Silkworm (*Bombyx mori*; ABY81279.1), AKH I Anopheles (*Anopheles gambiae*; XP_001689190.1), AKH Cockroach (*Periplaneta Americana*; AAV41425.1), AKH I Yellow Fever Mosquito (*Aedes aegypti*; XP_001655817.1), AKH Planthopper (*Nilaparvata lugens*; BAO00932.1), AKH I Tsetse Fly (*Glossina morsitans*; AEH25941.1), Crz Western Honey Bee (*Apis mellifera*; NP_001012981.1), Crz Giant Honey Bee (*Apis dorsata*; XP_006616128.1), Crz Leafcutter Bee (*Megachile rotundata*; XP_003706036.1), Crz Fruit Fly (*Ceratitis capitata*; XP_004533703.1), Crz Jonah Crab (*Cancer borealis*; [65]), Crz Gladiator (*Lobatophasma redelinghuysense*; B3A096.1) for comparison. Identical residues are shaded in red, residues with at least 50% identity are shaded in black and residues with 50% similarity are shaded in gray. Amino acid positions are numbered according to ap-AKH.
(PDF)
