# Supplementary material for: Localization and Functional Characterization of a Novel Adipokinetic Hormone in the Mollusk, Aplysia californica
Source: PLoS One. 2014 Aug 27;9(8):e106014. doi: 10.1371/journal.pone.0106014 (PMC4146582; doi:10.1371/journal.pone.0106014)
Supplement: Figure S1 — Alignment of amino acid sequences of AKH and Crz. Amino acid sequences of ap-AKH (Aplysia californica; AFN66119.1) aligned with: AKH Limpet (Lottia gigantea; AMQO01003719.1), AKH I Silkworm (Bombyx mori; ABY81279.1), AKH I Anopheles (Anopheles gambiae; XP_001689190.1), AKH Cockroach (Periplaneta Americana; AAV41425.1), AKH I Yellow Fever Mosquito (Aedes aegypti; XP_001655817.1), AKH Planthopper (Nilaparvata lugens; BAO00932.1), AKH I Tsetse Fly (Glossina morsitans; AEH25941.1), Crz Western Honey Bee (Apis mellifera; NP_001012981.1), Crz Giant Honey Bee (Apis dorsata; XP_006616128.1), Crz Leafcutter Bee (Megachile rotundata; XP_003706036.1), Crz Fruit Fly (Ceratitis capitata; XP_004533703.1), Crz Jonah Crab (Cancer borealis; [65]), Crz Gladiator (Lobatophasma redelinghuysense; B3A096.1) for comparison. Identical residues are shaded in red, residues with at least 50% identity are shaded in black and residues with 50% similarity are shaded in gray. Amino acid positions are numbered according to ap-AKH. (PDF) [file pone.0106014.s001.pdf]

	1	-	2	3	4	5	6	7	8	9	10	
AKH Aplysia	pQ	-	I	H	F	S	P	D	W	G	T	amide
AKH Limpet	pQ	-	I	H	F	S	P	T	W	G	S	amide
AKH I Silkworm	pQ	-	L	T	F	T	P	G	W	G	Q	amide
AKH I Anopheles	pQ	-	L	T	F	T	P	A	W	-	-	amide
AKH Cockroach	pQ	-	L	T	F	T	P	N	W	-	-	amide
AKH I Yellow Fever Mosquito	pQ	-	L	T	F	T	P	S	W	-	-	amide
AKH Planthopper	pQ	-	V	N	F	S	P	N	W	-	-	amide
AKH I Tsetse Fly	pQ	-	L	T	F	S	P	G	W	-	-	amide
Crz Western Honey Bee	pQ	T	F	T	Y	S	H	G	W	T	N	amide
Crz Giant Honey Bee	pQ	M	F	T	Y	S	H	G	W	T	N	amide
Crz Leafcutter Bee	pQ	T	F	Q	Y	S	H	G	W	T	N	amide
Crz Fruit Fly	pQ	T	F	Q	Y	S	H	G	W	T	S	amide
Crz Jonah Crab	pQ	T	F	Q	Y	S	R	G	W	T	N	amide
Crz Gladiator	pQ	T	F	H	Y	S	Q	G	W	T	N	amide
